# Endoscopic Image 2 Hours after PuraStat® Application: A Case of Achieving Hemostasis Using PuraStat® for Postgastric Lesion Biopsy Bleeding after Hemostatic Clips Failed

**DOI:** 10.1155/2023/5620348

**Published:** 2023-07-26

**Authors:** Yoshitsugu Misumi, Kouichi Nonaka, Maiko Kishino

**Affiliations:** Department of Digestive Endoscopy, Tokyo Women's Medical University Hospital, 8-1, Kawada-chou, Shinjuku-ku, Tokyo 162-8666, Japan

## Abstract

PuraStat® (3D Matrix, Tokyo, Japan) is a novel, self-assembling peptide hemostatic hydrogel that can be used endoscopically. Hemostasis can be physically obtained by covering bleeding points; however, there are no reports of how long PuraStat remains in the upper gastrointestinal tract. Herein, we report a case wherein esophagogastroduodenoscopy (EGD) was performed 2 hours after PuraStat application. A 73-year-old man underwent EGD for evaluation of lesions in the posterior wall of the stomach. A biopsy was then performed on the gastric lesions; however, massive bleeding occurred. A hemostatic clip was used to stop bleeding but failed; primary hemostasis was obtained by applying PuraStat. EGD performed 2 hours later to determine whether the patient could be discharged revealed that the white-turning PuraStat gel remained firmly in the applied area, confirming complete hemostasis. PuraStat is a hemostatic agent capable of physical hemostasis that reliably remains in the stomach even after a few hours of use and, thus, may replace some conventional hemostasis methods.

## 1. Introduction

Gastric lesion biopsy may cause massive bleeding with or without antithrombotic therapy [1]. When spontaneous hemostasis cannot be achieved, drug spraying, local injection, coagulation, and clipping can be utilized [2]; however, cases exist in which hemostasis remains challenging even with conventional methods. PuraStat is a novel, self-assembling peptide hemostatic hydrogel [3] that can be used endoscopically. Peptide molecules in PuraStat are neutralized by contact with body fluids such as blood or when electrolytes such as sodium or potassium are supplied to form fibers and become peptide hydrogels that cover bleeding points to stop bleeding physically [4]. Hemostatic efficacy for various gastrointestinal bleeding has been reported [5–7]; however, there are no reports on the kinetics of PuraStat in the upper gastrointestinal tract. Here, we report a case in which PuraStat was useful for hemostasis of bleeding after a biopsy of a gastric lesion that was difficult to stop with a hemostatic clip, and the status of PuraStat was confirmed in the stomach a few hours after its use.

## 2. Case Presentation

**Figure 1:**
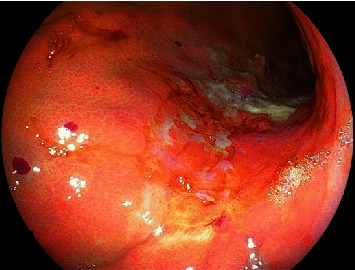
Early gastric cancer not indicated for endoscopic treatment is found on the posterior wall of the stomach.

**Figure 2:**
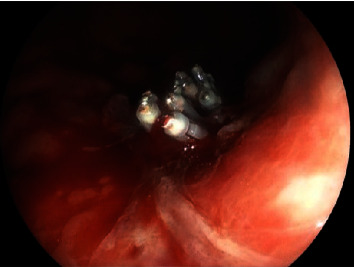
Since several hemostatic clips are used, identifying the bleeding points is difficult.

**Figure 3:**
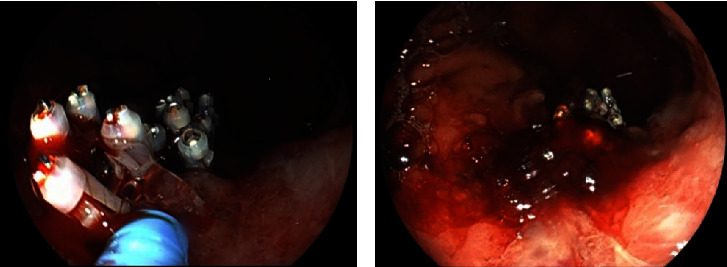
(a) A dedicated catheter is used to apply PuraStat to the approximate bleeding site. (b) Primary hemostasis is confirmed.

**Figure 4:**
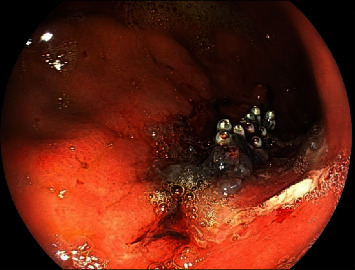
Endoscopic findings 2 hours after PuraStat application. The whitened gel remains intact, and complete hemostasis is achieved.

An asymptomatic 73-year-old man with a suspected gastric lesion, suspected to be gastric cancer by esophagogastroduodenoscopy (EGD), was referred to our hospital. The patient had no remarkable past medical history or physical examination findings and had not taken any antithrombotic medications. EGD was performed to obtain a detailed evaluation of the posterior wall lesion, which revealed that the lesion was not suitable for endoscopic treatment (Figure 1). A biopsy was performed for a definitive diagnosis, and a few days later, it revealed a differentiated adenocarcinoma. Subsequently, pulsatile bleeding occurred from the biopsied site, resulting in massive hemorrhage. Spontaneous hemostasis at the biopsied site was not achieved even after more than 5 minutes. We attempted to achieve hemostasis using clips, but the bleeding point shifted as more clips were added, which resulted in multiple hemostatic clips (Figure 2). Subsequently, the bleeding point could not be identified. A dedicated catheter was used to apply a total of 5 ml PuraStat over the approximate bleeding site (Figure 3(a)). Primary hemostasis was confirmed (Figure 3(b)). An EGD re-examination was performed 2 hours after the procedure to determine whether the patient could be discharged. Although we rinsed the gel with a water jet, the white-turning PuraStat gel remained firmly in the applied area, confirming that complete hemostasis had been achieved (Figure 4). The hemoglobin concentration, which consistently remained around 13 g/dl in regular blood tests, did not show a significant decrease to 12.9 g/dl in a blood sample taken fourteen days after the EGD procedure was performed.

## 3. Discussion

This case has confirmed that PuraStat remains reliable in the stomach for several hours after application in clinical practice. Since PuraStat is composed of peptides, it will not completely decompose and become a foreign substance or source of infection [8]. However, no clinical reports exist on how long it remains in the upper gastrointestinal tract. The hemostasis mechanism of PuraStat is said to be physical compression of the bleeding point [4], but if it does not remain in the digestive tract for a fixed period, there is no compression, and sufficient hemostasis may not be obtained. In this patient, PuraStat remained in the stomach 2 hours after use and sufficiently acted as a hemostatic agent. In addition, identifying bleeding points was difficult. However, since PuraStat is a gel material that can be applied extensively, hemostasis was obtained by applying it to the approximate area around the bleeding point. Failure of hemostasis with a hemostatic clip for colonic diverticular hemorrhage or hemorrhagic duodenal ulcer may result in a similar situation, and hemostasis with PuraStat may be an option. In addition, hemostasis with coagulation or clip methods may cause strong fibrosis in the submucosal layer. Since severe submucosal fibrosis is known to interfere with endoscopic resection [9], PuraStat may be particularly useful for hemostasis of postbiopsy bleeding in lesions that will require future endoscopic treatment, such as endoscopic submucosal dissection. In conclusion, PuraStat is a hemostatic agent that can remain in the stomach for a minimum of two hours after its use and can physically stop bleeding. Therefore, it may replace some conventional hemostasis methods.

## Data Availability

The data that support the findings of this study are available on request from the corresponding author.
